# Predicting Very Early-Stage Breast Cancer in BI-RADS 3 Lesions of Large Population with Deep Learning

**DOI:** 10.3390/jimaging11070240

**Published:** 2025-07-15

**Authors:** Congyu Wang, Changzhen Li, Gengxiao Lin

**Affiliations:** Cheeloo College of Medicine, Shandong University, Jinan 250012, China

**Keywords:** breast cancer, ultrasound, BI-RADS 3, early-stage malignancy, deep learning

## Abstract

Breast cancer accounts for one in four new malignant tumors in women, and misdiagnosis can lead to severe consequences, including delayed treatment. Among patients classified with a BI-RADS 3 rating, the risk of very early-stage malignancy remains over 2%. However, due to the benign imaging characteristics of these lesions, radiologists often recommend follow-up rather than immediate biopsy, potentially missing critical early interventions. This study aims to develop a deep learning (DL) model to accurately identify very early-stage malignancies in BI-RADS 3 lesions using ultrasound (US) images, thereby improving diagnostic precision and clinical decision-making. A total of 852 lesions (256 malignant and 596 benign) from 685 patients who underwent biopsies or 3-year follow-up were collected by Southwest Hospital (SW) and Tangshan People’s Hospital (TS) to develop and validate a deep learning model based on a novel transfer learning method. To further evaluate the performance of the model, six radiologists independently reviewed the external testing set on a web-based rating platform. The proposed model achieved an area under the receiver operating characteristic curve (AUC), sensitivity, and specificity of 0.880, 0.786, and 0.833 in predicting BI-RADS 3 malignant lesions in the internal testing set. The proposed transfer learning method improves the clinical AUC of predicting BI-RADS 3 malignancy from 0.721 to 0.880. In the external testing set, the model achieved AUC, sensitivity, and specificity of 0.910, 0.875, and 0.786 and outperformed the radiologists with an average AUC of 0.653 (*p* = 0.021). The DL model could detect very early-stage malignancy of BI-RADS 3 lesions in US images and had higher diagnostic capability compared with experienced radiologists.

## 1. Introduction

Breast cancer ranks among the most prevalent malignancies affecting women [1]. Its global incidence is steadily increasing and currently represents 24.5% of all newly diagnosed malignancies in women [2]. Early detection through breast cancer screening has the potential to boost the 5-year net survival rate by 15% and slash the mortality rate by 40% [3]. Diagnostic approaches for breast cancer encompass mammography, ultrasound (US), magnetic resonance imaging (MRI), and histopathological examination. Notably, ultrasound (US) screenings exhibit superior potential and sensitivity compared to mammography, particularly in enhancing the detection accuracy of early-stage and invasive cancers within dense and younger breast tissue. Consequently, it has become the primary method for early breast cancer screening in China [4,5,6,7].

The breast imaging reporting and data system (BI-RADS) classification is used to describe the breast lesions of US images from BI-RADS 1 to BI-RADS 5 and to identify appropriate follow-up and management of patients [8]. The category of BI-RADS 3 defines most probably benign lesions with malignancy risk less than 2%, and the American College of Radiology (ACR) guidelines state that such lesions can be managed with short-interval imaging follow-up (usually at 6, 12, and 24 months), rather than immediate biopsy [9]. Recent institution-level studies, however, show that the incidence of malignancy among the BI-RADS 3 category has been increasing, and the incidence of breast cancer became more than 4.9% by ultrasound in this category [10]. The misclassification of malignant lesions as BI-RADS 3 leads to potential risks of poor patient outcomes, such as a loss of treatment opportunities or delayed treatment [11].

We hypothesized that there are subtle but informative cues on US images that may not be discernible by ultrasound doctors or simple volume-of-density measurements [12], but DL models can make use of these cues to rate the malignancy risk of patients. There is a significant amount of DL-based work focusing on the early diagnosis of breast lesions in mammography, MRI, and US images (mainly focusing on BI-RADS 4 and BI-RADS 5 US images) [13,14]. Specifically, Ribli et al. [15] proposed a model based on Faster R-CNN to classify malignant and benign tumors in mammograms without radiologist intervention. Adachi et al. [16] employed RetinaNet as the CNN architecture for breast cancer detection in MRI images. However, this method was constrained by a small dataset and failed to detect invasive ductal carcinoma near the axilla. Similarly, Wu et al. [17] trained a CNN model to detect lesions in MRI images and demonstrated that single-sequence MRI at multiple time points provided sufficient information for CNN models to effectively identify malignant lesions. For US images, Becker et al. [18] trained a CNN model to differentiate between benign and malignant lesions, achieving an AUC of 0.84. Ciritsis et al. [19] further extended this work by employing a CNN model to classify BI-RADS 1-2, BI-RADS 3, and BI-RADS 4-5 lesions.

However, most existing studies have primarily focused on BI-RADS 4 and 5 lesions, which contain a considerable number of positive (malignant) and negative (benign) samples, leaving the accurate differentiation of high-risk malignant lesions among BI-RADS 3 cases an unresolved challenge. This difficulty arises from their inherent characteristics. Malignant cases often exhibit imaging features indistinguishable from benign morphology, while the extreme class imbalance caused by low malignancy prevalence further compounds the issue. These factors, combined with the limited availability of confirmed malignant samples, critically hinder DL models’ ability to learn robust discriminative features, making it difficult to improve classification accuracy for BI-RADS 3 lesions [20,21].

To address the class imbalance in BI-RADS 3 ultrasound images, an intuitive approach is to use sampling methods, like bootstrapping, to balance positive and negative samples. However, previous studies have shown that when the sample size is very small, such sampling methods contribute little to model performance [22]. This is because the dataset contains repeated samples, which do not provide additional information to the model. This limitation has motivated us to propose a novel double-channel deep learning (DL) model called Early Breast Cancer Viewer (EBCV) trained by knowledge distillation (KD)-enhanced transfer learning [23]. The model was pre-trained on the dataset, including a large number of unknown categories of BI-RADS malignant US images, and then transferred to the dataset, which was composed of BI-RADS 3 lesions. In transfer learning, the KD technique was used to enable the EBCV model as a student model and to learn more features from another dataset consisting of B-mode images through the teacher model, which was a single-channel model trained on the public dataset. In this way, our EBCV model can analyze US images to identify BI-RADS 3 high-risk malignant lesions and recommend immediate biopsy for confirmation.

In addition, data from different centers reflect the diversity of geographic regions, ethnicities, equipment, and operational standards. Different centers may use imaging equipment from various brands or models, leading to variations in the quality and features of the collected images. A model trained on data from a single center may perform well initially, but it could underperform when tested on external datasets, potentially due to overfitting. Therefore, using a multicenter dataset is essential to assess whether the model has good generalization ability, ensuring stable performance across different environments.

**Figure 1:**
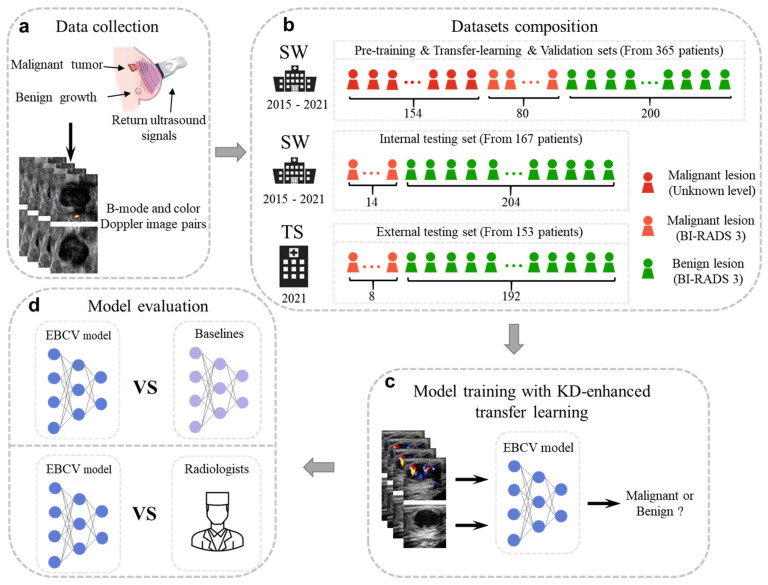
The overview of this study. (**a**) The conventional US diagnosis, where patients with BI-RADS 3 lesions were asked to undergo a 6-month follow-up. (**b**) The overview of the datasets we used. (**c**) Training the EBCV model by KD-enhanced transfer learning. (**d**) The methods we used to evaluate the performance of the proposed EBCV model.

In this retrospective cohort study, we included a total of 532 patients who were diagnosed as BI-RADS 3 by senior ultrasound doctors at Southwest Hospital (SW, located in Chongqing, southwest of China) and Tangshan People’s Hospital (TS, located in Hebei Province, northern China). We trained the proposed model and evaluated its performance on the primary dataset that was collected by SW, which is split into a pre-training set, a transfer learning set, a validation set, and an internal testing set. Moreover, we evaluated the generalization and robustness of our model across an independent dataset that was collected by TS and compared the diagnostic capabilities of the proposed model with those of six radiologists in the identification of BI-RADS 3 malignant lesions on the external testing dataset. Figure 1 shows the overview of the study.

In summary, this study has three contributions as follows:

(1) We developed a DL model for predicting very early-stage breast cancer in ultrasound (US) images and proposed a novel transfer learning approach to enhance the model’s performance.
(2) We employed multicenter datasets to validate the model’s cross-center generalization ability across different center-specific datasets.
(3) We conducted a comparison of our model’s diagnostic capabilities for early-stage breast cancer with those of six experienced radiologists using an external test dataset, affirming its clinical value. This comprehensive evaluation highlights the practical utility of the proposed model.

## 2. Materials and Methods

### 2.1. Study Population

This retrospective study was approved by the ethics committee of the First Affiliated Hospital of Army Medical University [No. (B) KY202264]; the requirement for patient informed consent was waived. We collected imaging data from 6578 patients at Southwest Hospital (SW) in Chongqing, China, from January 2015 to December 2021. We excluded 5999 patients who did not undergo biopsy or were followed up for less than three years. Of the remaining 579 patients, 9 were either 18 years old or of unknown age, 2 had malignant phyllodes tumors or other malignancies of non-mammary origin, were pregnant, or were found to have other medical issues, 18 were diagnosed with breast cancer or lobular carcinoma in situ with clinical signs of a non-mass accompanied by bloody nipple discharge, and 18 had insufficient-pixel images or had no B-mode or color Doppler images.

After excluding these patients, we used a dataset consisting of 652 lesions from 532 patients with biopsy-confirmed lesions or three years of follow-up. This primary dataset was divided into four sets: pre-training, transfer learning, validation, and internal testing sets. The pre-training set included images of biopsy-confirmed malignant (*n* = 154) and benign (*n* = 138) lesions. The transfer learning set included biopsy-confirmed BI-RADS 3 malignant lesions (*n* = 64) and benign lesions (*n* = 46). The validation set included biopsy-confirmed malignant (*n* = 16) and benign (*n* = 16) lesions of BI-RADS 3. The testing set included BI-RADS 3 malignant (*n* = 14) and benign lesions (*n* = 204).

**Figure 2:**
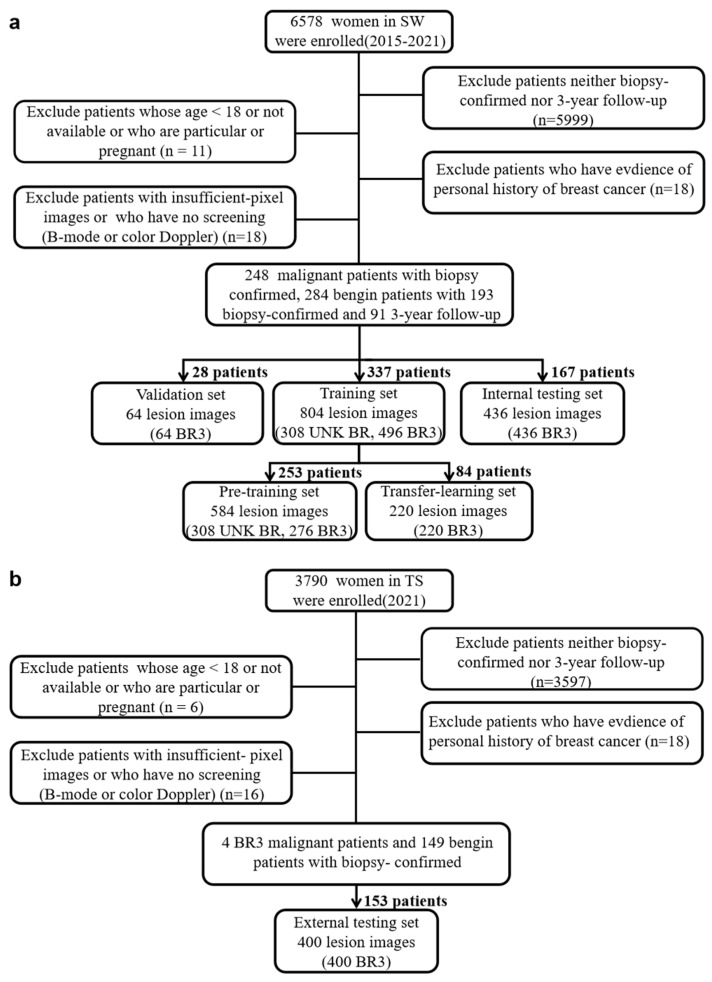
(**a**) The data workflow of this study. Due to the characteristics of retrospective investigation, multi-modal US images were not completely preserved and/or annotations were not integrally labeled in some lesions. (**b**) To employ the US image datasets from two medical centers, our EBCV model was developed and internally validated based on available US images. UNK BR: unknown BI-RADS category. BR3: BI-RADS 3.

In order to evaluate the generalization ability and the clinical practicability of the proposed model, we composed an external testing dataset and conducted a performance comparison between the EBCV model and six experienced radiologists on it. That was selected from a total of 3790 patients. Specifically, the external testing dataset consisted of 200 BI-RADS 3 lesions (8 malignant and 192 benign) from 153 patients collected by Tangshan People’s Hospital (TS) in 2021. The data workflow of both the primary dataset and the external test dataset is depicted in Figure 2. Due to the paucity of malignant BI-RADS 3 lesions, we employed a public dataset from the Kaggle platform, which included benign lesions (*n* = 445) and malignant lesions (*n* = 210) from 600 patients (without BI-RADS categories) in ages between 25 and 75 years old [24], to ensure the generalization ability of the model in the transfer learning phase.

### 2.2. Imaging Interpretation and Lesion Segmentation

**Figure 3:**
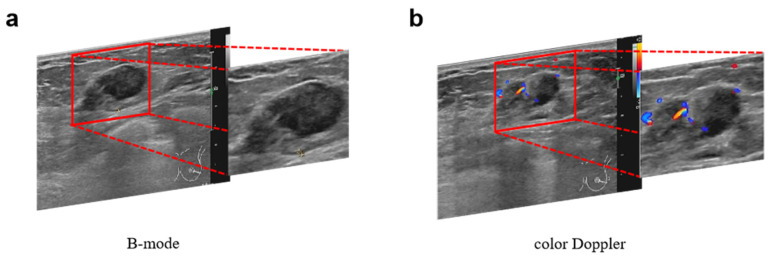
Lesion segmentation in US images of a 43-year-old woman who was diagnosed with fibroadenoma in the initial examination, but with invasive carcinoma confirmed by biopsy. (**a**) The B-mode US images. (**b**) The color Doppler US images, in which red indicates blood flow toward the transducer and blue indicates flow away from the transducer.

The details of the devices from which we collected US images of breast lesions are provided in Appendix A.4, Table A1. More specifically, image interpretation was performed by radiologists with an average of 20 years of clinical experience in breast US imaging. The 5th edition of the BI-RADS category was applied as a reference for feature assessment [25]. The suspicious lesion area in US images was manually segmented by the same radiologists who performed image interpretation and have an average of 20 years of clinical experience using the open-source ImageJ 1.53t software https://github.com/imagej (accessed on 15 March 2024). All segmented regions included a full lesion area, as illustrated in Figure 3.

### 2.3. Model Development

**Figure A1:**
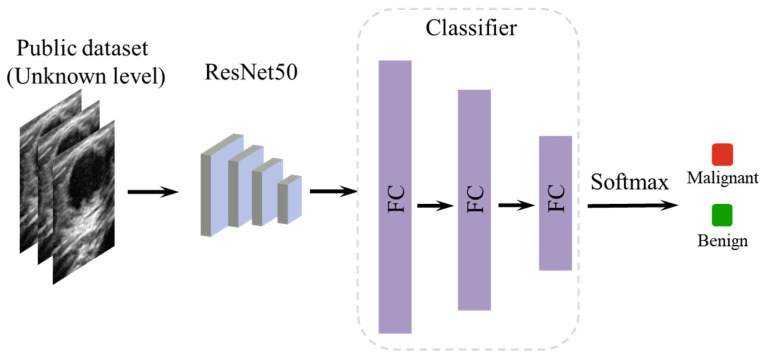
The structure of the teacher model. The teacher model was trained on a public dataset.

**Figure A2:**
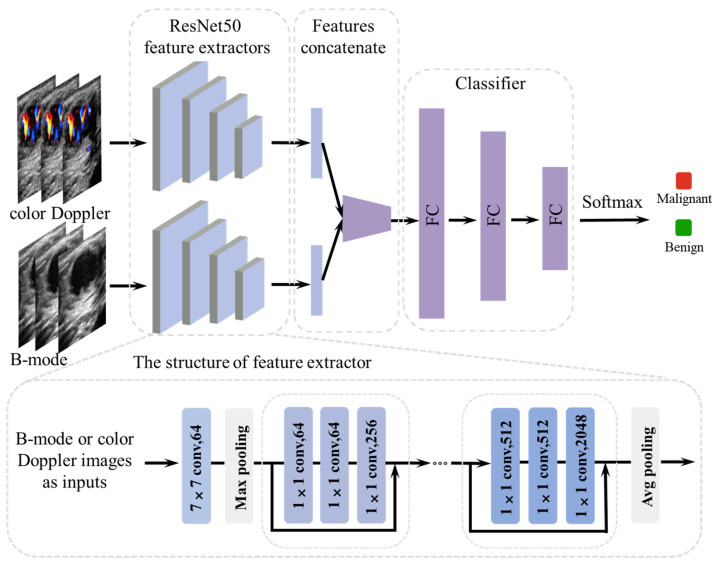
The conceptual architecture of the EBCV model in this study. The EBCV model consisted of two independent ResNet50 (pre-trained on ImageNet), a feature concatenation layer, three fully connected layers, and a softmax function. In addition, a dropped unit (with a probability of 0.3) was added after the second fully connected layer to prevent overfitting.

Before constructing the EBCV model, we conducted a comparative study to select the optimal backbone network (see Section 3.2 for details). The experimental results on our internal testing dataset confirmed that ResNet50 outperformed other candidate networks. Hence, it was chosen as the backbone network for all channel-mode models. Considering the limited number of malignant BI-RADS 3 lesions in our primary dataset, we first built a single-channel model (see Appendix A.4, Figure A1) with the inputs from the public dataset available on the Kaggle platform that consists of (no BI-RADS were categorized) B-mode US images as the teacher model (see Appendix A.1). After that, we constructed a double-channel model named EBCV with the inputs of both B-mode US images and color Doppler BI-RADS 3 US images in the primary dataset on the basis of the pre-trained teacher model. The EBCV model with two independent learning branches was designed to extract features from both pre-processed B-mode and color Doppler US images (the pre-processing details are shown in Appendix A.2), and the details of the EBCV model are depicted in Appendix A.4, Figure A2.

**Figure 4:**
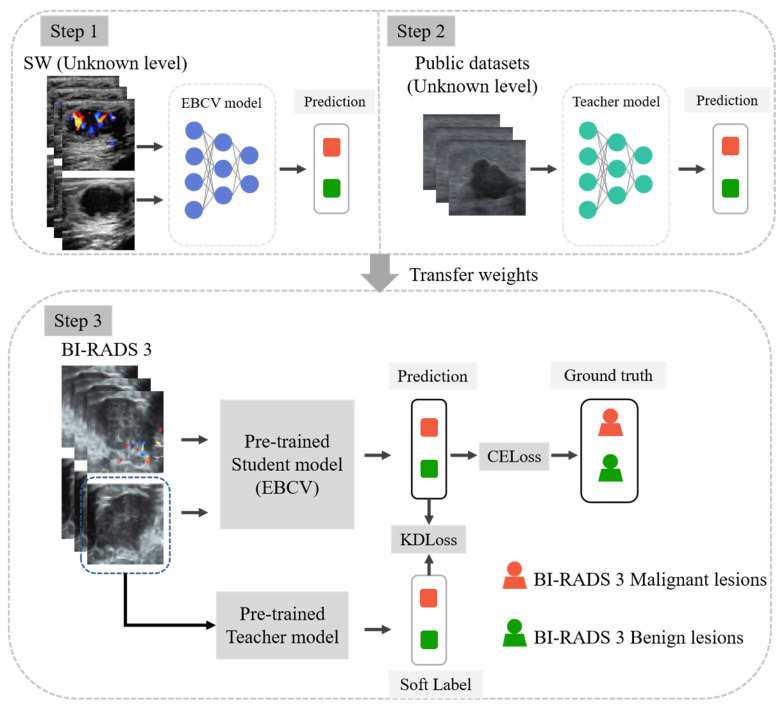
The details of KD-enhanced transfer learning; the teacher model was pre-trained on the public dataset with only unknown BI-RADS level B-mode images. CELoss: cross-entropy loss. KDLoss: knowledge distillation loss, where we used the KL divergence loss.

In this study, we utilized transfer learning to address the issue of insufficient malignant lesions of BI-RADS 3. The EBCV model was pre-trained on a dataset containing unknown BI-RADS levels of malignant lesions. In the transfer learning phase, we fine-tuned the model on a dataset that exclusively contained BI-RADS 3 malignant lesions, and two ResNet50 feature extractors in the model were frozen. To enhance the effectiveness of transfer learning, we employed knowledge distillation by utilizing a single-channel model as the teacher, pre-trained on a public dataset to capture its inherent information. In the third step, B-mode ultrasound images were fed into both the pre-trained teacher model and the student model, while color Doppler ultrasound images were input exclusively into the student model. In addition to the standard cross-entropy loss, we introduced a Kullback–Leibler (KL) divergence loss to align the student model’s predictions with the soft labels generated by the teacher model (as illustrated in Figure 4). Consequently, the EBCV model, serving as the student, benefited from features derived not only from the transfer learning dataset but also from the public dataset through guidance from the teacher model, achieving better generalization ability. More details about KD in the transfer learning phase are provided in Appendix A.3.

### 2.4. Comparison of Predictive Models

We proposed three baselines to verify the effectiveness of the proposed EBCV model, including a single-channel model trained on B-mode images, a single-channel model trained on color Doppler images, and a double-channel model trained by the conventional transfer learning (CTL) method. In addition to the validation dataset, we used the internal testing dataset to compare the performance of the EBCV model with other baselines.

### 2.5. Performance Comparison Between the EBCV Model and Radiologists

To further validate the diagnostic capability of the EBCV model in clinical practice, we invited six radiologists who have more than 20 years of clinical experience analyzing both B-mode and color-Doppler images, and the TS testing dataset was used to compare its performance with the six radiologists. The tests were conducted using the same cropped images by the model and radiologists on a web-based rating platform. All six radiologists were aware that the patients in the external testing dataset had undergone biopsy, but they were blinded to the pathology reports of patients in the dataset.

### 2.6. The Interpretability of Model

To improve the interpretability of the model and mitigate the black-box property of the EBCV model, we used Grad-CAM to generate heatmaps to show the interpretability of the model [26]. These heatmaps embodied the location of the model’s focus on the US images, associated with the model prediction results. We obtained the heatmaps by applying Grad-CAM to the last convolutional layer of each ResNet50 in the EBCV model. Further details on Grad-CAM can be found in Appendix A.4.

### 2.7. Evaluation Metrics and Statistical Analysis

We evaluated the performance of our proposed model by analyzing the receiver operating characteristic curve (ROC), area under the receiver operating characteristic curve (AUC), sensitivity, specificity, negative predictive value (NPV), and positive predictive value (PPV). We used the DeLong test to compare the difference in the AUC while using different baselines and our model [27]. We performed all statistical analyses in MedCalc Statistical Software version 18.11.3 (MedCalc Software bvba; www.medcalc.org). A two-sided *p*-value test (*p* < 0.05) was considered to validate the results as statistically significant.

## 3. Results

### 3.1. Patient Characteristics

After applying the aforementioned exclusion criteria (see Section 2.1), 685 patients with 852 lesions from SW and TS hospitals remained eligible for the study. A total of 434 (50.9%) lesions were used for the pre-training, transfer learning, and validation of the model, and 418 (49.1%) lesions were used for testing. Demographic data and patient characteristics are summarized in Table 1.

### 3.2. Performance Comparison with Different Backbones

To develop a high-performance model, we evaluated four commonly used backbone networks: VGG16 [28], SENet50 [29], EfficientNet [30], and ResNet50 [31]. Each network was pre-trained on B-mode ultrasound images and subsequently tested on our internal dataset. As shown in Table 2, ResNet50 demonstrated superior performance compared to the other architectures. Based on these results, we selected ResNet50 as the backbone network for our EBCV model.

### 3.3. Performance of the Proposed Model

**Figure 5:**
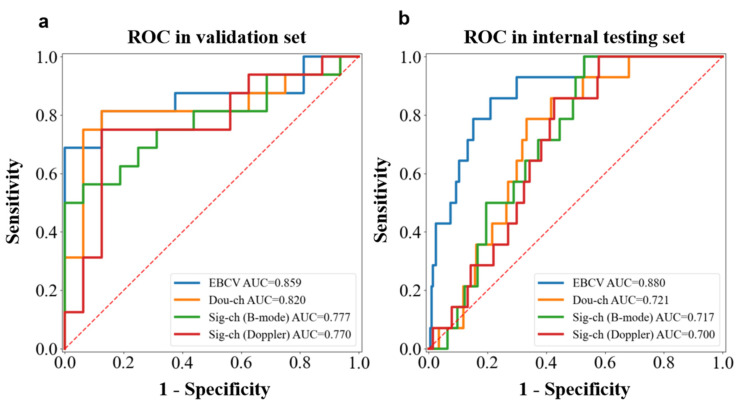
The ROC analysis of the EBCV model, single-channel model (B-mode), single-channel model (color Doppler), and double-channel model (CTL) in the validation dataset (**a**) and internal testing dataset (**b**).

**Figure A3:**
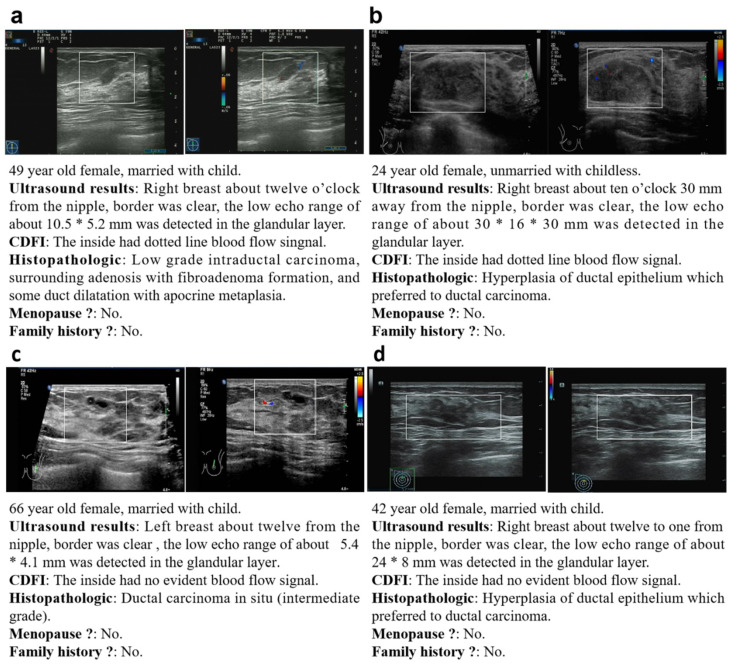
Diagnostic reports for four false-negative cases predicted by the EBCV model from the internal testing set (**a**–**c**) and the external testing set (**d**).

In the validation dataset, the EBCV model exhibited better discrimination capability than the single-channel models trained by only B-mode or color Doppler images, as well as the double-channel model, which was trained by the traditional transfer learning method (refer to Figure 5a). In the internal testing dataset, we observed a similar model performance (Figure 5b). The AUC of our model was significantly higher than that of the other baselines (*p* < 0.05). The experimental results indicated that our KD-enhanced transfer learning method has a better generalization ability than the CTL method. The sensitivity, specificity, and accuracy of the EBCV model in the internal testing set were 0.785 (11 of 14), 0.833 (170 of 204), and 0.830 (181 of 218), respectively. Based on these results, the EBCV model could potentially identify eleven malignant cases, which were diagnosed as benign by doctors in initial examination, including six invasive carcinomas, four intraductal carcinomas, and one carcinoma in situ. Note that among the three false-negative predictions made by the EBCV model, two cases are adenosis and one case is fibroadenoma (Appendix A.4, Figure A3a–c). A detailed comparison between the validation and internal testing sets is shown in Table 3.

### 3.4. Comparison of Diagnostic Efficiency Between Our Model and Radiologists

**Figure 6:**
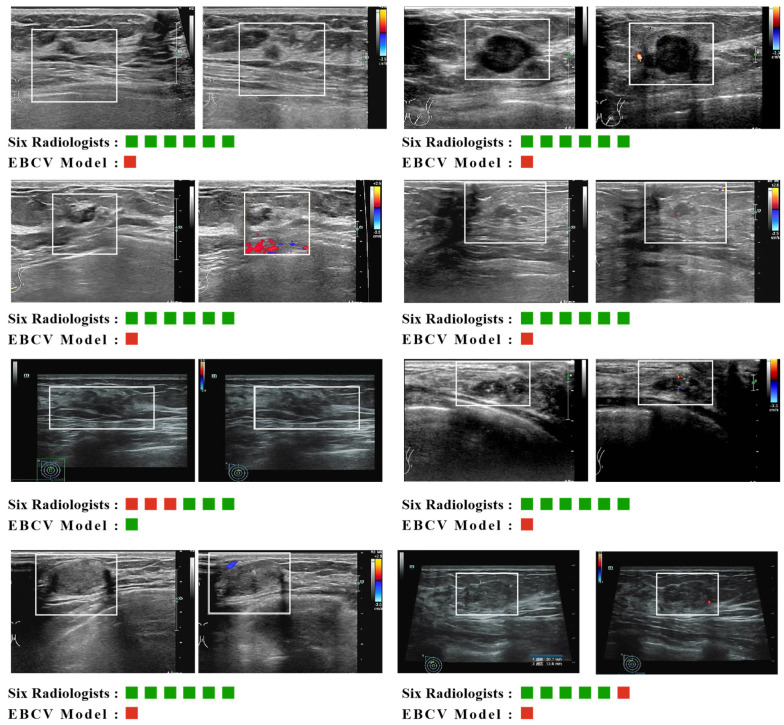
The diagnostic results of the EBCV model and six radiologists for the malignant lesions in the external testing dataset. Red squares denote malignant diagnoses and green squares denote benign diagnoses. The white boxes in the US images represent where the lesions are.

**Figure 7:**
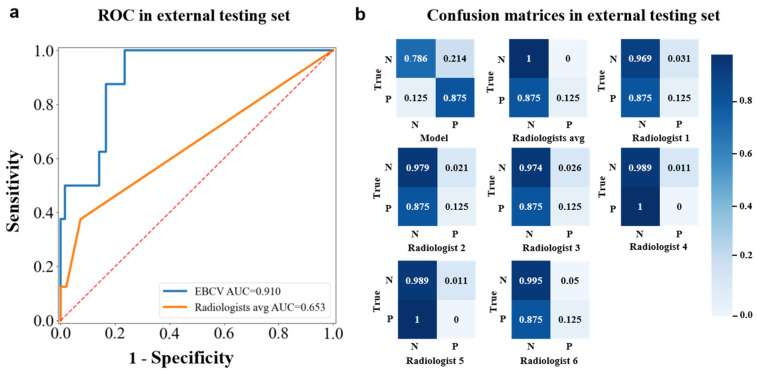
Comparison of performance in predicting between the EBCV model and radiologists in the external testing dataset. (**a**) The ROC analysis in the external testing set. (**b**) The confusion matrices of predictions of the EBCV model and radiologists. The value of each row in the confusion matrix was divided by the total number of this category.

We compared the performance of our model with six breast radiologists in diagnosing breast malignancies on the external testing dataset. In this study, each radiologist provided a clear diagnosis of benign or malignant for the given lesion. That is to say, the malignant probability for the lesion is either 0 or 1, so we could not calculate the AUC for each radiologist. To compare with the radiologists, we calculated the average AUC of six radiologists as follows. If a radiologist correctly identifies a malignant lesion, the probability of malignancy for that lesion will be increased by 1/6. Our model predicted that a total of seven malignancies could be identified in time, out of which four were invasive carcinoma and three were carcinoma in situ. One false-negative prediction made by the EBCV model was diagnosed by US doctors as adenosis (Appendix A.4, Figure A3d), and more details about the diagnostic comparison of the model and radiologists for malignant lesions of the external set are shown in Figure 6. Note that for the only one false-negative diagnosis made by the EBCV model, there were merely three radiologists who made a correct diagnosis. The results indicated that our model has a higher diagnostic ability for BI-RADS 3 malignancies than the radiologists. Table 4 presents the sensitivity, specificity, NPV, PPV, and AUC of the EBCV model and the radiologists. Figure 7a represents the results of the ROC analysis, and Figure 7b shows the relevant confusion matrices. It is worth mentioning that the proposed EBCV model exhibits a lower true negative rate compared to the radiologists. This observed discrepancy stems primarily from two interrelated factors inherent in the model’s design and clinical context. First, the EBCV model is specifically optimized to maximize sensitivity for detecting BI-RADS 3 malignancies, a design priority that inevitably increases false positives by classifying certain benign lesions with malignant characteristics as positive—a clinically justified trade-off given the critical importance of early cancer detection. Second, radiologists demonstrate an inherent diagnostic conservatism when evaluating BI-RADS 3 lesions due to their subtle imaging features, resulting in higher true negative rates but potentially at the expense of sensitivity. Importantly, the marginally reduced true negative performance of our model represents a clinically acceptable compromise to achieve its primary objective of early malignancy identification, which remains paramount in breast cancer screening scenarios. This performance characteristic aligns with established clinical priorities where the timely detection of malignancies outweighs the consequences of false positives in this diagnostic context.

### 3.5. Interpretation of the DL Model

The blood flow images displayed by color Doppler US images can vividly and intuitively show the direction, velocity, and characteristics of blood flow. In general, the richer the blood flow, the higher the likelihood of malignancy, as the blood supply provides the necessary conditions for tumor growth. In addition, malignant tumors are more likely to spread due to rapid cell division, leading to irregular shapes.

**Figure 8:**
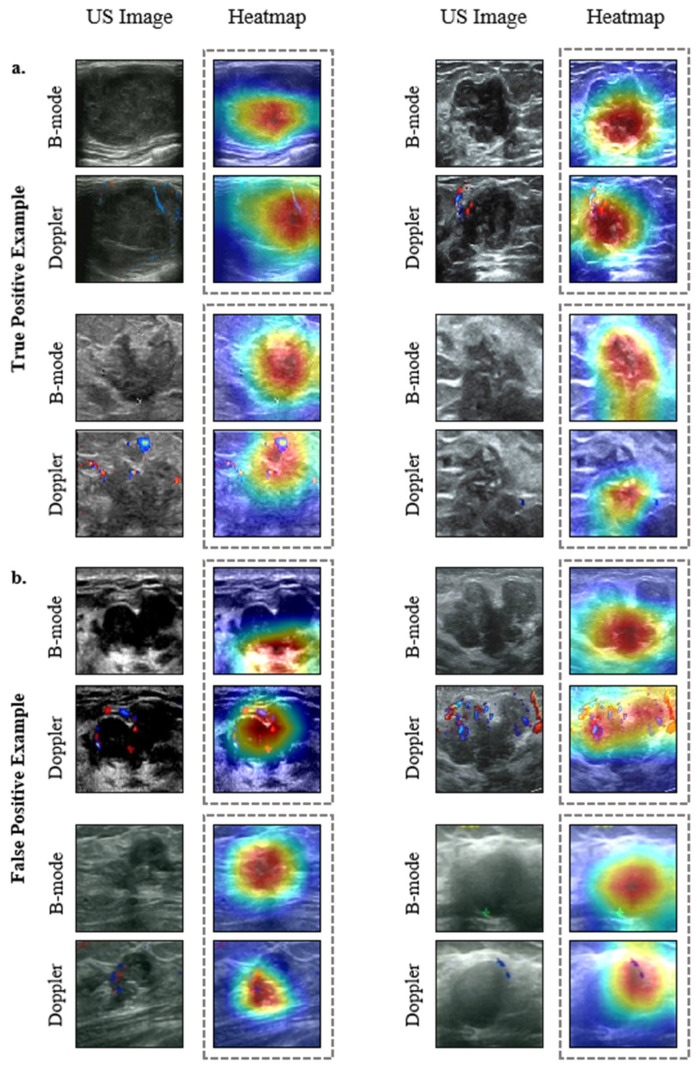
Representative examples of the heatmaps. (**a**) Heatmaps of one true-positive case. (**b**) Heatmaps of one true-negative case. The red color highlights where the model paid attention.

**Figure A4:**
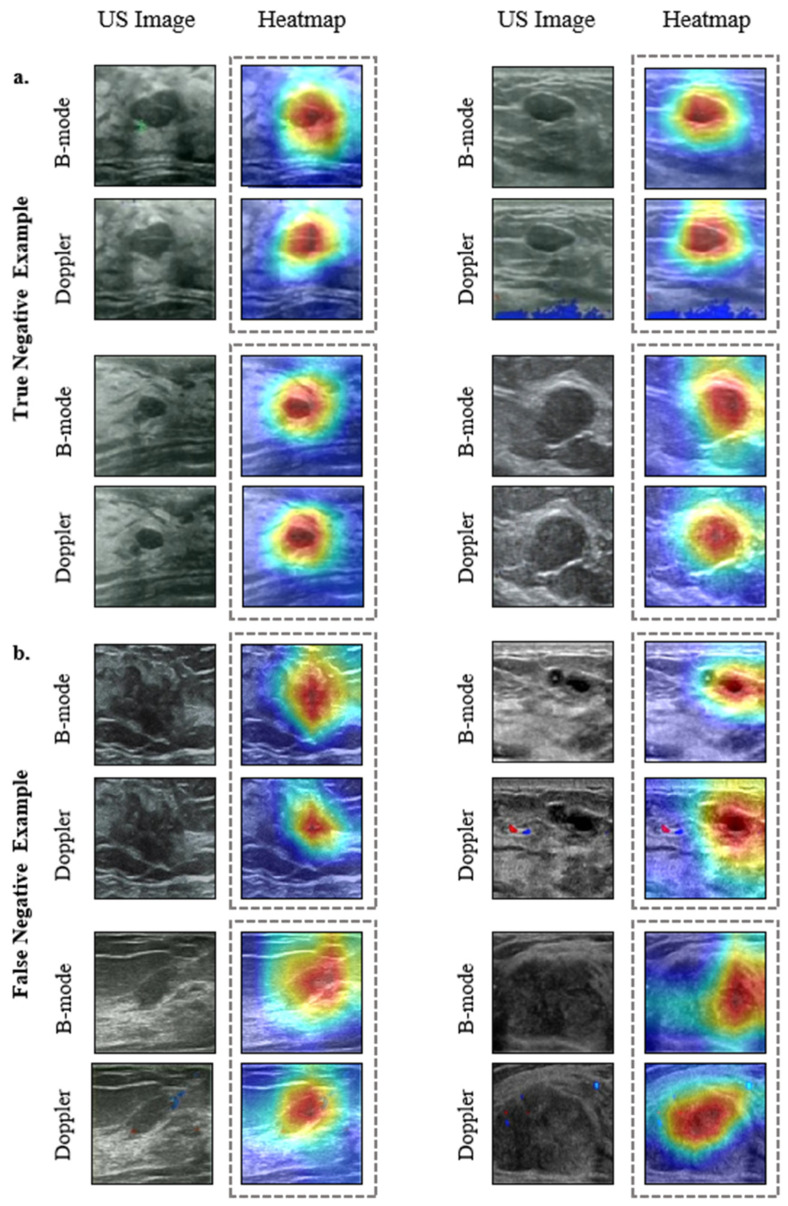
Representative examples of the heatmaps. (**a**) Heatmaps of one true-negative case. (**b**) Heatmaps of one false-negative case. The red color highlights where the model paid attention.

To better understand the regions in ultrasound images where our diagnostic model focused and increased its interpretability, we used Grad-CAM to extract regions of attention. The results showed that the heatmap signals generated by the model were not located in the same regions on the B-mode or color Doppler US images. The EBCV model was sensitive to blood flow signals around and inside of tumors that are inconspicuous on the color Doppler images, while it paid more attention to the contour of the edge of the benign lesions on the B-mode images. It detected strong signals in 24 false-positive cases (24 benign lesions in the nipple and areola area). Some traditional cases are provided in Figure 8, and other cases can be seen in Appendix A.4, Figure A4. These results confirm that the EBCV model has the potential to identify subtle vascular features that might otherwise be overlooked, particularly in cases where blood flow patterns serve as critical indicators of malignancy. Additionally, the model effectively leverages detailed morphological characteristics from B-mode images to differentiate between benign and malignant cases, demonstrating its capability to achieve higher diagnostic accuracy.

## 4. Discussion

In this study, we developed a double-channel model named EBCV on the basis of KD-enhanced transfer learning. We employed a single-channel model as a teacher model to assist the double-channel model in identifying malignancies from BI-RADS 3 lesions, with the support of knowledge distillation. Our results indicated that compared with the double-channel model trained by the CTL method with AUCs of 0.820 (95% CI: 0.645–0.933) and 0.721 (95% CI: 0.656–0.779), in the validation and internal testing datasets, the EBCV model yielded AUC values of 0.859 (95% CI: 0.691-0.956) and 0.880 (95% CI: 0.829–0.920). They suggested that the EBCV model presented a comparable ability in identifying early-stage malignancy of BI-RADS 3 with other baselines but had a higher specificity, which means it could discriminate malignancy efficiently. Furthermore, we showed that the diagnostic ability of our model for early breast cancer was significantly better than that of six radiologists, with an AUC of 0.910 (95% CI: 0.861–0.945) compared to 0.653 (95% CI: 0.583–0.719) in the external testing dataset. Although the EBCV model identified more false-positive cases (41 of 192 benign cases) compared with the six radiologists, it could diagnose malignancy more efficiently, with a favorable sensitivity of 0.875 (seven of eight malignant cases). We suggest that accurate prediction of BI-RADS 3 malignancy is vital for a large Chinese population, as it can help find BI-RADS 3 malignant patients and prevent deterioration and thus improve survival probability, with timely treatment.

Breast cancer progression is closely linked to tumor vascularization, with studies demonstrating a positive correlation between vessel density, tumor size, and pathological severity [32]. Early-stage breast cancer, in particular, is characterized by increased vascular density and disorganized vessel distribution [33], a finding further supported by evidence showing that malignant lesions typically exhibit centralized vascularization, in contrast to the uniform vascular patterns observed in benign lesions [23]. Notably, Doppler imaging overlaid on B-mode ultrasound has revealed detectable vascularization in 99% of malignant lesions compared to only 4% of benign cases [24,25]. In this study, our proposed EBCV model demonstrated a unique ability to detect subtle blood flow signal changes in color Doppler images of early breast cancer (Figure 8). Specifically, the heatmaps of predicted malignant tumors displayed enhanced vascular signals and increased blood flow velocity, which are crucial for improving diagnostic accuracy in BI-RADS 3 patients. We hypothesize that the EBCV model captures signals originating from microvessels rather than structural features in B-mode images, as no similar signals were detected in B-mode analysis. This phenomenon may be attributed to two factors: (1) scattered microvascular signals with insufficient pixel density to display conventional color-coded flow (red/blue) or (2) slow microvascular blood flow below the velocity threshold required for color visualization. These findings suggest that the model has the ability to resolve subvisual hemodynamic features that are undetected by standard radiologist interpretation, representing its primary diagnostic advantage and offering a potential breakthrough in early malignancy detection.

**Figure A5:**
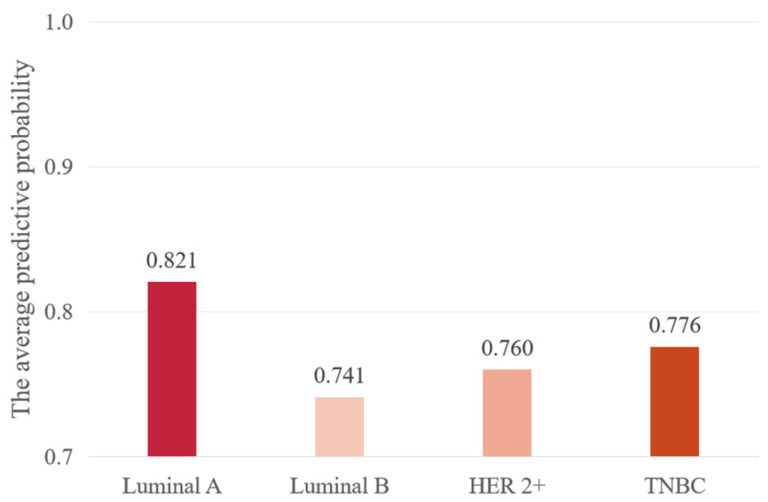
The average predictive probability of different molecular subtypes made by the EBCV model on the testing datasets (including SW and TS testing datasets).

Moreover, our findings indicate that the EBCV model can offer distinctive predictions across diverse molecular subtypes of early-stage breast cancer. Notably, it exhibits the highest predictive probability for luminal A, which stands as the most prevalent molecular subtype, typically constituting 40–50% of breast cancer cases [34]. In contrast, the EBCV model demonstrates a relatively diminished predictive probability for luminal B. We speculated that this phenomenon might be related to the inherent attributes of luminal B, which is renowned for its marked heterogeneity, characterized by substantial disparities in molecular attributes and clinical manifestations among individual patients. This inherent heterogeneity poses a formidable challenge for the model to learn the shared characteristics of this particular subtype, thus leading to diminished predictive probabilities. The results of different molecular subtypes are provided in Appendix A.4, Figure A5.

In fact, DL algorithms have been applied in clinical diagnosis of breast cancer, lung cancer, prostate cancer classification, and screening [21,35,36,37,38,39], which proves the high diagnostic efficiency and capability of the DL models to reduce the workload of doctors. Specifically, Liu et al. [40] proposed a DL model to predict microcalcifications in BI-RADS 4, and the relevant results demonstrated its validity and clinical practicability [40]. Nastase et al. [41]. presented a method-based transfer learning method, which integrates the features (extracted from the tissue inside the tumor, the lesion, and the surrounding tissue) with US images to improve malignant lesion identification.

We emphasize that our work makes the following two contributions. First, our model specifically focuses on early-stage breast lesions classified as BI-RADS 3, which often exhibit benign features but may harbor malignant potential. In clinical practice, a considerable proportion of breast cancer patients are initially diagnosed as BI-RADS 3 based on ultrasound examinations due to the subtle or ambiguous imaging characteristics of these lesions. However, over time, some of these patients develop clinical abnormalities, and further diagnostic tests, such as biopsy or follow-up imaging, eventually confirm the presence of malignant tumors. This delayed diagnosis can lead to missed opportunities for early intervention and potentially worsen patient outcomes. With the proposed EBCV model, we aim to address this critical gap by enabling earlier detection of malignancies within the BI-RADS 3 category. By leveraging advanced feature extraction and classification techniques, our model can identify subtle patterns indicative of malignancy that may be overlooked by conventional diagnostic methods. This capability allows for the timely identification of high-risk patients, facilitating earlier intervention and improving the likelihood of successful treatment. Ultimately, the EBCV model has the potential to enhance clinical decision-making, reduce the rate of missed diagnoses, and improve patient prognosis in cases where BI-RADS 3 lesions progress to malignancy.

Secondly, to rigorously evaluate the robustness and generalizability of our proposed model, we recruited a larger cohort of patients from two independent medical centers with diverse demographic and clinical characteristics. This multicenter approach ensures that our model is tested on a more representative and heterogeneous dataset, reflecting real-world variability in patient populations and imaging protocols. By validating the model’s performance across different centers, we aimed to assess its cross-center generalization ability, which is a critical factor for successful clinical deployment.

Additionally, compared with existing large models, such as LLaVA-Med [42], the proposed model requires significantly fewer computational resources (see Appendix A.4, Figure A2), making it highly suitable for deployment in resource-limited environments. For instance, in rural or remote areas where medical resources are often scarce and access to advanced diagnostic tools is limited, our model can serve as a cost-effective and efficient solution. Its low resource requirements enable it to run on standard, widely available hardware without the need for high-performance computing infrastructure. This ensures that healthcare providers in underserved regions, such as countryside clinics, can leverage the model to improve diagnostic accuracy and provide timely care to patients. By bridging the gap in medical resource disparities, our model has the potential to enhance healthcare equity and accessibility, particularly for populations in low-resource settings. More importantly, the EBCV model has the potential to be extended to the diagnosis of early malignant lesions in other organs, such as bone, liver, and thyroid, which utilize reporting and data systems similar to BI-RADS.

However, our study has several limitations. Firstly, although we included a relatively large dataset from Southwest Hospital and Tangshan People’s Hospital, the ratio between benign and malignant samples of the internal testing set and the external testing set is not consistent with that of the training set. Nevertheless, the proportion of malignant samples in testing sets was close to that of malignancy in BI-RADS 3, making our test results applicable to individuals with early-stage malignancies of breast lesions. Secondly, we cannot currently explain the false-positive cases obtained in this study, but it is found that most false positive cases (24 among the 34 false-positive cases) in the internal testing set embody intense blood flow signals; we speculate that blood flow signals have some correlation with false-positive cases, and further studies are needed.

## 5. Conclusions

In conclusion, we propose a deep learning (DL) model with knowledge distillation (KD)-enhanced transfer learning for analyzing BI-RADS 3 breast lesions. The proposed EBCV model demonstrates strong performance in extracting morphological features, enabling effective and objective image analysis for early breast cancer detection. Notably, it achieves an AUC of 0.910, along with a sensitivity of 0.875 and a specificity of 0.786, significantly outperforming experienced radiologists (average AUC: 0.653). These results underscore the model’s potential to enhance diagnostic accuracy and provide reliable decision support for clinicians.

## Figures and Tables

**Table 1:** Clinical characteristics of patients in the pre-training, transfer learning, validation, and testing sets. The primary sets (including pre-training, transfer learning, validation, and internal test sets) were collected by Southwest Hospital of China. The external testing dataset was collected by Tangshan People’s Hospital. Note that all the lesion information was determined using existing screening and diagnostic reports.

Specifications	Pre-Training and Transfer Learning and Validation Set	Internal Testing Set	External Testing Set
Patients (685 patients from 2 centers; 252 patients developed cancers)			
Age			
<30	164 (44.9%)	48 (28.7%)	44 (28.8%)
30~49	183 (50.1%)	113 (67.7%)	104 (68.0%)
50~69	17 (4.7%)	6 (3.6%)	5 (3.2%)
≥70	1 (0.3%)	0	0
Diagnostic methods			
Biopsy	258 (70.7%)	87 (52.1%)	153 (100%)
Follow-up	107 (29.3%)	80 (47.9%)	0
Lesions (852 lesions and 256 malignant lesions, including 102 BI-RADS 3 malignant lesions)			
Lesion size (mm^2^)			
<5	18 (4.1%)	29 (13.3%)	19 (9.5%)
5~9.9	103 (23.7%)	126 (57.8%)	112 (56.0%)
10~19.9	209 (48.2%)	60 (27.5%)	63 (31.5%)
≥20	104 (24.0%)	3 (1.4%)	6 (3.0%)
Lesion width (mm)			
<5	122 (28.1%)	133 (61.0%)	112 (56.0%)
5~9.9	224 (51.6%)	78 (35.8%)	78 (39.0%)
10~19.9	83 (19.1%)	7 (3.2%)	10 (5.0%)
≥20	5 (1.2%)	0	0
Aspect ratio			
≥1	7 (1.6%)	2 (0.9%)	5 (2.5%)
<1	427 (98.4%)	216 (99.1%)	195 (97.5%)
Boundary			
Circumscribed	375 (86.4%)	203 (93.1%)	169 (84.5%)
Others	59 (13.6%)	15 (6.9%)	31 (15.5%)
Morphology			
Regular	389 (89.6%)	210 (96.3%)	178 (89%)
Others	45 (10.4%)	8 (3.7%)	22 (11%)
Blood flow spectrum			
Pulsating	7 (1.6%)	0	3 (1.5%)
Others	427 (98.4%)	218 (100%)	197 (98.5%)
Malignant type (biopsy result)			
Invasive carcinoma	113 (48.3%)	6 (42.9%)	4 (50%)
Carcinoma in situ	93 (39.7%)	7 (50%)	4 (50%)
Mucinous Adenocarcinoma	18 (7.7%)	0	0
Others	10 (4.3%)	1 (7.1%)	0

**Table 2:** A comparison of the prediction results in the internal testing datasets with different backbone networks. (SEN, sensitivity; SPE, specificity; AUC, area under the receiver operating characteristic curve; Acc, accuracy; NPV, negative predictive value; PPV, positive predictive value; CI, confidence interval.)

Models	SEN	SPE	ACC	AUC (95% CI)	NPV	PPV
VGG16	0.571 (8/14)	0.451 (92/204)	0.459 (100/218)	0.638 (0.530–0.691)	0.862 (100/116)	0.064 (8/125)
SENet50	0.643 (9/14)	0.475 (97/204)	0.486 (106/218)	0.674 (0.551–0.729)	0.912 (115/126)	0.076 (9/118)
EfficientNet	0.714 (10/14)	0.480 (98/204)	0.491 (107/218)	0.681 (0.613–0.737)	0.919 (114/124)	0.086 (10/117)
ResNet50	0.714 (10/14)	0.505 (103/204)	0.518 (113/218)	0.717 (0.652–0.776)	0.970 (127/131)	0.090 (10/111)

**Table 3:** A comparison of the prediction results in the validation and internal testing datasets. (SEN, sensitivity; SPE, specificity; AUC, area under the receiver operating characteristic curve; Acc, accuracy; NPV, negative predictive value; PPV, positive predictive value; CI, confidence interval.)

	Models	SEN	SPE	ACC	AUC (95% CI)	NPV	PPV
Validation	Sig-ch (B-mode)	0.75 (12/16)	0.563 (9/16)	0.656 (21/32)	0.777 (0.596–0.905)	0.692 (9/13)	0.632 (12/19)
	Sig-ch (Doppler)	0.75 (12/16)	0.50 0 (8/16)	0.625 (20/32)	0.770 (0.587–0.899)	0.667 (8/12)	0.600 (12/20)
	Dou-ch (CTL)	0.812 (13/16)	0.750 (12/16)	0.781 (25/32)	0.820 (0.645–0.933)	0.800 (12/15)	0.765 (13/17)
	EBCV model	0.812 (13/16)	0.875 (14/16)	0.842 (27/32)	0.859 (0.691–0.956)	0.824 (14/17)	0.867 (13/15)
Testing	Sig-ch (B-mode)	0.714 (10/14)	0.505 (103/204)	0.518 (113/218)	0.717 (0.652–0.776)	0.970 (127/131)	0.090 (10/111)
	Sig-ch (Doppler)	0.857 (12/14)	0.466 (89/204)	0.463 (101/218)	0.700 (0.635–0.760)	0.978 (89/91)	0.945 (12/127)
	Dou-ch (CTL)	0.785 (11/14)	0.534 (109/204)	0.550 (120/218)	0.721 (0.656–0.779)	0.976 (121/124)	0.104 (11/106)
	EBCV model	0.785 (11/14)	0.833 (170/204)	0.830 (181/218)	0.880 (0.829–0.920)	0.983 (170/173)	0.244 (11/45)

**Table 4:** A comparison of our deep learning model and six radiologists in the external testing dataset. (SEN, sensitivity; SPE, specificity; AUC, area under the receiver operating characteristic curve; ACC, accuracy; NPV, negative predictive value; PPV, positive predictive value; CI, confidence interval.)

Models	SEN	SPE	ACC	AUC (95% CI)	NPV	PPV
Radiologist 1	0.125 (1/8)	0.969 (186/192)	0.935 (187/200)	N/A	0.963 (186/193)	0.143 (1/7)
Radiologist 2	0.125 (1/8)	0.979 (188/192)	0.945 (189/200)	N/A	0.969 (188/194)	0.200 (1/5)
Radiologist 3	0.125 (1/8)	0.974 (187/192)	0.940 (188/200)	N/A	0.964 (187/194)	0.167 (1/6)
Radiologist 4	0 (0/8)	0.989 (190/192)	0.950 (190/200)	N/A	0.960 (190/198)	0
Radiologist 5	0 (0/8)	0.989 (190/192)	0.950 (190/200)	N/A	0.960 (190/198)	0
Radiologist 6	0.125 (1/8)	0.995 (191/192)	0.960 (192/200)	N/A	0.965 (191/198)	0.500 (1/2)
Radiologists’ Avg	0.125 (1/8)	1.00 (192/192)	0.965 (193/200)	0.653 (0.583–0.719)	0.965 (192/199)	1.00 (1/1)
EBCV Model	0.875 (7/8)	0.786 (151/192)	0.790 (158/200)	0.910 (0.861–0.945)	0.993 (151/152)	0.146 (7/48)

## Data Availability

The datasets used in the current study are available from the corresponding author upon reasonable request and with permission of Southwest Hospital of Third Military Medical University. The retrospective datasets on a web-based rating platform are available at http://birads.ssdlab.cn/admin (accessed on 14 August 2024). The code generated during the current study is available from http://112.74.95.118:8888/down/cMEFo5mYNVxH (accessed on 14 March 2025).

## Abbreviations

BI-RADS	Breast imaging reporting and data system
US	Ultrasound
MRI	Magnetic resonance imaging
DL	Deep learning
CNN	Convolutional neural network
CTL	Conventional transfer learning
KD	Knowledge distillation
EBCV	Early breast cancer viewer
ROC	Receiver operating characteristic curve
NPV	Negative predictive value
PPV	Positive predictive value
AUC	Area under the receiver operating characteristic curve
SEN	Sensitivity
SPE	Specificity
ACC	Accuracy
CI	Confidence interval

## Appendix A

### Appendix A.1. The Development of the Teacher Model

The single-channel model, which we used as the teacher model in the transfer learning phase, was trained on the public data from the Kaggle platform to learn the features of malignancy to teach the double-channel model (Figure A1). We trained the model for 60 epochs with a batch size of 24. The initial learning rate was set to 0.0005, and the weight decay was 0.0005. The Adam optimizer was used to update the model parameters.

### Appendix A.2. The Data Pre-Process and Implementation Details

To enrich the dataset and improve image quality so that it was suitable for model training, we pre-processed the datasets. First, we denoted regions of interest based on the regions originally annotated by the examining radiologists, removed irrelevant information, and resized the B-mode and color Doppler images to 224 × 224 pixels. Then, we randomly cropped the images to 200 × 200 pixels and implemented the following augmentation strategies: horizontal flipping (probability, 0.5), image blurring, contrast-limited adaptive histogram equalization, random brightness/contrast/saturation alteration, random removal of 5 × 5 pixel regions (probability, 0.3), random rotation (–9° and 9°), and random shearing (−10° and 10°). Finally, we resized the images to 180 × 180 pixels for the input of the model. The experiments were implemented using the Pytorch (version 1.8) framework and Python (version 3.9) environment on a Windows 10 64× Intel 10700 CPU with 32GB RAM and an NVIDIA GeForce 3060 GPU with 12 GB of memory.

### Appendix A.3. The Detail of Transfer Learning Combining Knowledge Distillation

In the pre-training phase, we trained the model for 60 epochs with a batch size of 24. The initial learning rate was set to 0.0005, and the weight decay was 0.0005. The Adam optimizer was used to update the model parameters. In the transfer learning phase, the learning rate was reduced to 0.00005. In addition to the standard cross-entropy loss, we introduced *KL* divergence loss to match the prediction results of the single-mode model and our two-mode model. The *KL* divergence loss between them was calculated as follows:

$$KL(P||Q)=\sum_{i=1}^{N}P_{i}\log\left(\frac{P_{i}}{Q_{i}}\right)$$

where *P* was the prediction result of the student model and *Q* was the soft label generated by the teacher model. We hoped that the double-channel model as a student model could take the prediction result of the teacher model into consideration while carrying out transfer learning. After introducing the teacher model, our final loss function was as follows:

$$Loss=\lambda \times L_{KL}(prediction/\tau ,\;soft\;label/\tau )+L_{prediction}$$

$L_{KL}$ is the KL divergence loss, and the soft label denotes the prediction of the teacher model. $L_{prediction}$ is the loss with the ground truth. τ is a hyperparameter, which we set to 0.5. τ denotes a constant temperature parameter, which we set to 1.15.

### Appendix A.4. The Details of Grad-Cam

For class *c*, we first obtained the importance weight of the neurons ${\;a}_{k}^{c}$ by calculating the gradient of the scores $y^{c}$ of the feature maps $A^{k}$ of the last convolutional layer in the model, and then we performed the global average pooling as follows:

$$a_{k}^{c}=\frac{1}{Z}\sum_{i}\sum_{j}\frac{\partial y^{c}}{\partial A_{ij}^{k}}$$

where *Z* is the number of features of the associated feature map. We then applied a linear correction function (ReLU) to the weighted combination of these feature maps to generate the heatmap $h_{Grad-CAM}^{c}$ as follows:

$$h_{Grad-CAM}^{c}=ReLU(\sum_{k}a_{c}^{k}A^{k})$$

**Table A1:** US devices used to capture the dataset images. The US images (including B-mode and color Doppler) in the SW dataset and the external datasets were scanned by different Doppler US devices. There was no bias in terms of device preference for the training and validation datasets in this study.

Devices	Datasets from SW		Dataset from TS
Philips EPIQ7	434	218	-
GE E9	260	130	242
Siemens	86	42	-
Mindray R9	88	46	-
Philips EPIQ5	-	-	158
